# Factors Affecting Route Selection of Balloon-Assisted Enteroscopy in Patients with Obscure Gastrointestinal Bleeding: A KASID Multicenter Study

**DOI:** 10.3390/diagnostics11101860

**Published:** 2021-10-10

**Authors:** Dong Hoon Baek, Seonyeong Hwang, Chang Soo Eun, Seong Ran Jeon, Jinsu Kim, Eun Ran Kim, Dong-Hoon Yang, Hyun Joo Jang, Jong Pil Im, Soo Jung Park, Sung Hoon Jung

**Affiliations:** 1 Department of Internal Medicine, Pusan National University School of Medicine, Busan 49421, Korea; dhbeak77@gmail.com; 2 Department of Internal Medicine, Eunpyeong St. Mary’s Hospital, College of Medicine, The Catholic University of Korea, Seoul 03312, Korea; jcnyong@nate.com (S.H.); jinsu23@naver.com (J.K.); 3 Department of Internal Medicine, Hanyang University Guri Hospital, Hanyang University College of Medicine, Guri 11923, Korea; cseun@hanyang.ac.kr; 4 Department of Internal Medicine, Soon Chun Hyang University School of Medicine, Seoul 04401, Korea; 94jsr@hanmail.net; 5 Department of Internal Medicine, Samsung Medical Center, Sungkyunkwan University School of Medicine, Seoul 06351, Korea; er.kim@samsung.com; 6 Department of Internal Medicine, Asan Medical Center, University of Ulsan College of Medicine, Seoul 05505, Korea; dhyang@amc.seoul.kr; 7 Department of Internal Medicine, Hallym Medical Center, Hallym University College of Medicine, Seoul 14068, Korea; jhj1229@hallym.or.kr; 8 Department of Internal Medicine, Seoul National University College of Medicine, Seoul 03080, Korea; jp-im@hanmail.net; 9 Department of Internal Medicine, Yonsei University College of Medicine, Seoul 03722, Korea; sjpark@yuhs.ac

**Keywords:** obscure gastrointestinal bleeding, small bowel bleeding, enteroscopy, capsule endoscopy

## Abstract

Balloon-assisted enteroscopy (BAE) is an important diagnostic modality for ongoing obscure gastrointestinal bleeding (OGIB). However, it is difficult to determine the optimal insertion route. We retrospectively analyzed the records of patients with OGIB contained in a multicenter enteroscopy database of 1108 balloon-assisted enteroscopy (BAE) procedures (875 patients) to find out factors affecting BAE route selection in patients with OGIB. A total of 603 BAE procedures in 512 patients were investigated: there were 392 (65.0%) bidirectional and 211 (35.0%) unidirectional procedures. Overt OGIB was more frequent in the latter group (*p* = 0.024). Computed tomography (CT) was more frequently performed in the unidirectional group (*p* < 0.001). Capsule endoscopy and a small bowel barium study were performed more frequently in the bidirectional group (*p* < 0.001 and *p* = 0.039, respectively). Multivariate analysis showed that occult OGIB, capsule endoscopy and a small bowel barium study were independently associated with use of the bidirectional approach (*p* = 0.011, *p* = 0.013 and *p* = 0.046, respectively). Conversely, CT was associated with use of the unidirectional approach (*p* < 0.001). Conclusion: CT can aid the selection of an optimal insertion route in OGIB patients. However, capsule endoscopy and small bowel barium study are unhelpful.

## 1. Introduction

Obscure gastrointestinal bleeding (OGIB), until recently termed small bowel bleeding or potential small bowel bleeding, is relatively rare, accounting for 5–10% of all cases of gastrointestinal (GI) bleeding [1,2,3]. Most OGIB is caused by small bowel bleeding, The American College of Gastroenterology (ACG) recommends that OGIB be reclassified as small bowel bleeding [2]. Recently, various imaging modalities, including video capsule endoscopy (VCE), deep enteroscopy and radiographic imaging have been applied to the small bowel. The utility of these modalities depends on the extent of bleeding and the patient’s condition. Several guidelines have been proposed by various societies [2,4,5,6]. The Japanese guidelines propose that computed tomography (CT) should be considered before capsule endoscopy as the first-line procedure for small bowel evaluation. Device-assisted enteroscopy (DAE) is recommended for patients with persistent overt bleeding or who are OGIB-positive on capsule endoscopy or CT. DAE, including double-balloon [7], single-balloon [8], and spiral enteroscopy [9], enables simultaneous endoscopic tissue sampling and hemostasis. Although DAE is ideal for small bowel evaluation, the use of DAE is very limited given the long procedure times, technical difficulties and need for extensive equipment and several assistants. A recent study introduced a new form of enteroscopy; prototype single-balloon enteroscopy with passive bending and high force transmission that helps deep insertion into the small intestine [10], but the use of DAEs still has technical limitations. One systematic review of double-balloon endoscopy reported that serious complications included perforation, pancreatitis and bleeding, at a rate of 0.72% (95% confidence interval [CI] 0.56–0.90%) [11]. Thus, it is important to consider whether DAE is appropriate and to perform the procedure carefully. An appropriate insertion route is also important, but to the best of our knowledge no study has addressed this topic. Therefore, we aimed to identify factors affecting DAE route selection in OGIB patients by analyzing a large, multicenter enteroscopy database.

## 2. Materials and Methods

### 2.1. Patients and Study Design

We extracted data for OGIB patients who underwent enteroscopy from a multicenter, retrospective BAE registry. OGIB was defined as bleeding of unknown origin that persisted or recurred after negative initial or primary upper and lower endoscopy. We obtained data on demographic characteristics and factors affecting the BAE insertion route, including the OGIB type, initial insertion route, use of a unidirectional or bidirectional approach, final diagnosis and diagnostic modality. Patients were divided into unidirectional and bidirectional groups by the oral and/or anal approach. Data collection and analysis were approved by the institutional review board of each participating institution.

### 2.2. BAE

Enteroscopic examinations were performed using a double-balloon enteroscope (EN-450P5/20, T5/20; Fujinon Inc., Saitama, Japan) and a single-balloon enteroscope (SIF-Q180; Olympus, Tokyo, Japan), which are both available in South Korea. The insertion route was determined based on the clinical features, capsule endoscopy, CT and other imaging modalities. If the oral approach was used, patients fasted for 8–12 h before BAE; if the anal approach was used, patients ingested 2–4 L of polyethylene glycol-electrolyte lavage solution on the day before BAE. All procedures were performed with patients under conscious to deep sedation (established by the endoscopists) according to the sedation protocols of the various centers.

### 2.3. Definitions

The bidirectional approach was both oral and anal, while the unidirectional approach was oral or anal. We divided OGIB into overt and occult types. Overt OGIB was associated with the passage of visible blood (melena or hematochezia) from a bleeding source in the small bowel, while occult OGIB referred to iron-deficiency anemia with or without a positive fecal occult blood test. The final diagnosis was broadly classified as tumorous disease (benign or malignant tumors and polyposis, non-tumorous disease (vascular and inflammatory lesions), lesions of the stomach and duodenum, or lesions of the colon.

### 2.4. Statistical Analysis

The *t*-test and chi-squared test were used to compare data between the unidirectional and bidirectional groups. Variables that were significant, or showed a trend toward significance, in the univariate analysis were included in a multivariate (binary logistic regression) analysis. SAS software (ver. 9.0; SAS Institute, Cary, NC, USA) was used for all analyses. A *p*-value < 0.05 was considered to indicate statistical significance.

## 3. Results

### 3.1. Baseline Characteristics of the Study Subjects

**Figure 1:**
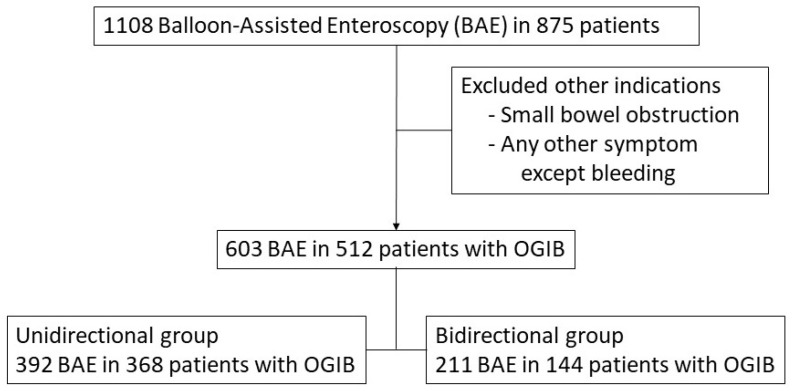
Flow diagram of the present study. BAE, balloon-assisted enteroscopy; OGIB, obscure gastrointestinal bleeding.

We reviewed a total of 1108 BAEs performed on 875 patients. Of these, 603 BAEs of 512 patients with OGIB were included in the final analysis (Figure 1). The unidirectional and bidirectional groups contained 392 (65.0%) and 211 (35.0%) BAEs, respectively. The mean patient age did not differ between the two groups (52.2 ± 18.5 vs. 49.4 ± 18.1 years, *p* = 0.731). Overt OGIB was more common in the unidirectional than bidirectional group (*p* = 0.024). The initial insertion route and final diagnosis did not differ between the two groups. Other patient characteristics are summarized in Table 1.

### 3.2. Diagnostic Modalities

The diagnostic modalities used before BAE included CT, capsule endoscopy, a barium study, a bleeding scan and an angiographic Meckel scan. CT was more frequently performed in the unidirectional group (*p* < 0.001). Capsule endoscopy and a small bowel barium study were more frequently performed in the bidirectional group (*p* < 0.001 and *p* = 0.039, respectively). The diagnostic modalities are listed in Table 2.

### 3.3. Factors Associated with the Bidirectional Approach

On univariate analysis, occult OGIB, low blood urea nitrogen (BUN) and creatinine levels, high protein and albumin levels, no CT examination, capsule endoscopy and a small bowel barium study were associated with the bidirectional approach. Multivariate analysis (Table 3) showed that occult OGIB was independently associated with the bidirectional approach. Of the diagnostic modalities, capsule endoscopy and a small bowel barium study were associated with the bidirectional approach (*p* = 0.013 and *p* = 0.046, respectively). Conversely, CT was associated with the unidirectional approach (*p* < 0.001). On univariate analysis, we found no significant correlation between the bidirectional approach and a low BUN or high albumin level.

## 4. Discussion

BAE yields tissue samples for diagnosis and allows endoscopic hemostasis in OGIB patients. The diagnostic rate is 60–80% and the treatment success rate 40–73% [12,13,14]. In 40–50% of patients, BAE findings influence the treatment strategy [15]. Recently, urgent BAE (<72 h) of OGIB patients has been reported to improve the diagnostic rate compared to non-urgent BAE (70% vs. 30%, *p* < 0.050); the treatment rate was also improved (54% vs. 15%, *p* < 0.001) [16]. However, BAE can cause various complications. The procedure-related mortality rate is 0.01% and care is required in these cases [11]. In addition, BAE takes a relatively long time and several medical personnel are needed, including nurses, assistant doctors and radiologists. Therefore, it is very important to determine the optimal insertion route.

We found that CT identified the optimal insertion route and the bidirectional approach became unnecessary. This supports the Japanese guideline, which states that CT is essential in the absence of a contra-indication such as renal failure or contrast allergy [5,17,18]. The American, European and Korean guidelines suggest that capsule endoscopy should be the first choice diagnostic modality [2,4,6]. Differences in the modalities used have been attributed to CT availability [18]. However, CT is quicker than capsule endoscopy and identifies tumors and bleeding foci. DAE combined with abdominal CT is a simple and effective method for the diagnosis of intestinal vascular malformation bleeding [19]. Therefore, CT should be performed to determine the BAE insertion route.

Capsule endoscopy and a small bowel barium study can also locate OGIB foci. The diagnostic rate and positive and negative predictive rates, of capsule endoscopy for OGIB are 60–83%, 94–97% and 83–100%, respectively. Recent studies have shown that the capsule transit time was useful for determining the DBE route [20,21]. This contrasts with our findings that capsule endoscopy and a small bowel barium study were associated with the bidirectional route. There are several possible explanations. First, BAE can be performed without prior capsule endoscopy in patients with overt OGIB. In such cases, the optimal insertion route can be selected by reference to the bleeding pattern (hematochezia or melena). We found that the optimal insertion route was determined more often in patients with overt than occult OGIB. Second, capsule endoscopy may serve as the first-choice diagnostic modality for occult OGIB, where it may be difficult to locate the bleeding focus. A recent study indicated that the failure rates of capsule endoscopy were 18.9%, 5.9% and 0.5% for small bowel tumors, vascular disease and ulcers, respectively [22], suggesting that it may be difficult to determine the optimal insertion route for BAE. Third, the small bowel barium studies had low diagnostic OGIB rates (3–17%) [23,24,25,26] and was, thus, not recommended for evaluation of OGIB [2].

Several limitations of this study should be addressed. First, it was a retrospective registry study. In addition, BAEs were performed by several endoscopists in various centers, so technical differences in BAE implementation were inevitable. However, that the study included a large number of OGIB BAEs from a large, multicenter enteroscopy database.

## 5. Conclusions

An optimal BAE insertion route in OGIB is important; CT is invaluable in this regard. Unlike previous studies, we found that capsule endoscopy and small intestinal imaging were unhelpful in selecting an optimal BAE insertion route. In patients with overt (in contrast to occult) OGIB, it may be easier to determine the optimal insertion route. Although CT availability varies among countries, CT scan is most commonly used than capsule endoscopy and maybe it is the most cost-effective initial imaging strategy. Thus, a country-specific prospective study of CT efficacy prior to BAE in OGIB patients is required.

## Figures and Tables

**Table 1:** Patient characteristics (Univariate analysis).

	Total (*n* = 603)	Unidirectional Group (*n* = 392)	Bidirectional Group (*n* = 211)	*p*-Value
Age		52.2 ± 18.5	49.4 ± 18.1	0.731
Sex				0.807
Male	379 (62.8)	245 (62.5)	134 (63.5)	
Female	224 (37.2)	147 (37.5)	77 (36.5)	
OGIB				0.024
Overt	528 (87.6)	352 (89.8)	176 (83.4)	
Occult	75 (12.4)	40 (10.2)	35 (16.6)	
Medical History				
DM	92 (15.3)	65 (16.6)	27 (12.8)	0.218
HTN	162 (26.9)	100 (25.5)	62 (29.4)	0.306
LC	28 (4.6)	21 (5.4)	7 (3.3)	0.256
ESRD	22 (3.7)	19 (4.9)	3 (1.4)	0.039
Crohn’s disease	19 (3.2)	12 (3.1)	7 (3.3)	0.864
Behcet disease	6 (1.0)	1 (0.3)	5 (2.4)	0.022
Medication				
Aspirin	99 (16.4)	68 (17.4)	31 (14.7)	0.401
Antiplatelet agent	35 (5.8)	26 (6.6)	9 (4.3)	0.236
Anticoagulant	21 (3.9)	12 (3.1)	9 (4.3)	0.442
Laboratory finding				
Hemoglobin(g/dL)		9.2 ± 2.6	9.0 ± 2.4	0.463
Platelet		231.6 ± 103.6	248.5 ± 99.8	0.059
BUN		19.1 ± 16.4	16.3 ± 13.8	0.036
Creatinine		1.2 ± 1.9	0.9 ± 0.4	0.001
Protein		5.8 ± 1.0	6.1 ± 1.0	0.004
Albumin		3.4 ± 0.7	3.5 ± 0.7	0.054
Initial				0.134
Insertion Route				
Oral approach	359 (59.5)	242 (61.7)	117 (55.5)	
Anal approach	244 (40.5)	150 (38.3)	94 (44.6)	
Final diagnosis				0.848
Tumorous	67 (11.1)	46 (11.7)	21 (10.0)	
Non-tumorous	515 (85.4)	333 (85.0)	182 (86.3)	
UGI	17 (2.8)	11 (2.8)	6 (2.8)	
Colon	4 (0.7)	2 (0.5)	2 (1.0)	

Values are presented as mean ± SD or *n* (%). OGIB, obscure gastrointestinal bleeding; DM, diabetes mellitus; HTN, hypertension; LC, liver cirrhosis; ESRD, end-stage renal disease; UGI, upper gastrointestinal track.

**Table 2:** Diagnostic modalities (univariate analysis).

	Total (*n* = 603) N (%)	Unidirectional Group (*n* = 392) N (%)	Bidirectional Group (*n* = 211) N (%)	*p*-Value
CT				<0.001
No	197 (32.7)	99 (25.3)	98 (46.5)	
Yes	406 (67.3)	293 (74.7)	113 (53.6)	
Capsule				<0.001
No	407 (67.5)	286 (73.0)	121 (57.4)	
Yes	196 (32.5)	106 (27.0)	90 (42.6)	
Barium study				0.039
(small bowel)				
No	393 (65.2)	267 (68.1)	126 (59.7)	
Yes	210 (34.8)	125 (31.9)	85 (40.3)	
Bleeding Scan				0.747
No	459 (76.1)	300 (76.5)	159 (75.4)	
Yes	144 (23.9)	92 (23.5)	52 (24.6)	
Angiography				0.186
No	448 (74.3)	298 (76.0)	150 (71.1)	
Yes	155 (25.7)	94 (24.0)	61 (28.9)	
Meckel scan				0.878
No	553 (91.7)	359 (91.6)	194 (91.9)	
Yes	50 (8.3)	33 (8.4)	17 (8.1)	

Values are presented as *n* (%). CT, computed tomography.

**Table 3:** Factors affecting DAE route selection in OGIB patients (Multivariate analysis).

	Estimated Value	Standard Error	Odds Ratio	95% CI	*p*-Value
Age	−0.002	0.006	0.075	0.988–1.009	0.784
Sex	0.087	0.202	0.184	0.734–1.622	0.668
OGIB (Occult)	0.704	0.278	6.433	1.174–3.485	0.011
CT	–0.923	0.203	20.590	0.267–0.592	<0.001
Capsule	0.506	0.204	6.137	1.112–2.477	0.013
Barium study (small bowel)	0.405	0.203	3.983	1.007–2.230	0.046
BUN	–0.008	0.007	1.310	0.977–1.006	0.252
Albumin	0.180	0.147	1.494	0.987–1.598	0.222

DAE, device-assisted enteroscopy; CI, confidence interval; OGIB, obscure gastrointestinal bleeding; CT, computed tomography.

## Data Availability

The datasets generated or analyzed during the current study are available from the corresponding author on reasonable request.
